# Dengue among suspected patients with dengue admitted at a tertiary level hospital in Mymensingh region of Bangladesh: A hospital-based epidemiological study

**DOI:** 10.1371/journal.pntd.0013047

**Published:** 2025-04-28

**Authors:** Md. Saiful Islam Khan, Md. Abu Sayem, Md. Mothashin, Md. Nurul Islam, Md. Golam Hossain

**Affiliations:** Health Research Group, Department of Statistics, University of Rajshahi, Rajshahi, Bangladesh; Colorado State University, UNITED STATES OF AMERICA

## Abstract

**Background:**

Dengue, a mosquito-borne disease predominantly found in tropical and subtropical countries like Bangladesh. The Aedes species particularly *Aedes aegypti* carry and transmit the dengue virus thus causing dengue fever irrespective of age, gender, race or religion. Limited studies on dengue in low endemic zone of Bangladesh are available. This study attempted to investigate factors influencing dengue among suspected patients with dengue in a tertiary level hospital at Mymensingh region of Bangladesh.

**Methods:**

A hospital-based cross-sectional study was conducted among 381 suspected patients with dengue admitted to Mymensingh Medical College Hospital, Mymensingh, Bangladesh from June 2023 to December 2023. A face to face interview was performed and there were no ignored cases in this study. Dengue infection was confirmed by a positive result of NS1 dengue antigen test if the blood sample taken within 7 days of the onset of fever, and IgM antibody test if the blood sample taken after 7th day of symptoms. The chi-square test and binary logistic regression model were used to determine the influencing factors of dengue using SPSS software (IBM version 25).

**Results:**

Most of the suspected patients came from urban environment (64.3%). The prevalence of dengue among suspected patients with dengue admitted at hospital was 74.3%, among them 45.9% and 28.3% were confirmed as dengue fever by NS1 antigen and IgM test respectively. Logistic regression model demonstrated that female had more likely to get dengue than male [aOR = 2.08, CI:1.09-3.93; p < 0.05]. Similarly, patients came from urban environment [aOR = 5.99, CI:3.09-11.64; p < 0.01], travel history to Dhaka in last two weeks preceding the survey [aOR = 11.21, CI:5.30-23.72; p < 0.01], participants did not use mosquito net during sleeping at day time [aOR = 2.74, CI:1.41-5.55; p < 0.01] and presence of water containers around the house [aOR = 12.00, CI: 5.69-25.29; p < 0.01] had higher chance to get dengue compared to their counterparts.

**Conclusion:**

More than 25% suspected patients were not identified as dengue patients. Some factors were identified as risk for dengue. A safe working and living environment, self and community awareness and planned urbanization can help to prevent breeding of mosquito larvae that transmit dengue virus thus causing dengue.

## Summary

Dengue is a growing concern and alarming public health problem in Bangladesh. Previously the dengue was endemic in Dhaka, Chattogram and Khulna divisions. But gradually it has spread throughout the country with higher incidence of deaths. Though, the government of Bangladesh advertising using Bangladesh television (BTV) channel about the sources of dengue, environment, transmissibility and availability of services and supports, people are still unaware about the associated factors thus causing dengue. On the other hand, government interventions such as destroying of breeding places and infected mosquitos, waste management and disposal, distribution of mosquito repellents, frequency of advertisement using most of the TV channels, accommodating optimum patients for both urban and rural community and overall health systems are not strong enough to prevent and control of dengue in Bangladesh. Furthermore, certain socio-demographic and behavioral factors are not being properly evaluated by government health wing. So, authors tried to extract the associated factors to provide recommendations to national and regional policy makers to develop awareness strategies and necessary actions to prevent and control of dengue in Mymensingh region, Bangladesh.

## Introduction

Dengue fever has considered as a substantial public health concern in Asia since 1950 [1]. Still it is a growing concern as favorable mosquito breeding temperature and environment in tropical and subtropical countries of Asian region [2]. Though the other regions such as Europe, America, Eastern Mediterranean are not free of dengue, South East Asian (ASEAN) countries account for more than half of the global burden of dengue. From 2015 to 2019 in the ASEAN region had seen a 46% growth in dengue cases, with Indonesia, Myanmar, and Thailand being some of the most highly endemic countries in the world [3]. South and Southeast Asian countries including Bangladesh, India, Pakistan, and Sri Lanka are at higher risk of dengue related mortality and morbidity due to rapid urbanization with overcrowded slums and inadequate infrastructure creates breeding grounds for Aedes mosquitoes [1,4].

In Bangladesh, the first official dengue outbreak occurred in 2000, with 5551 cases and 93 deaths [5]. Subsequently, an increasing trend was observed over time. More than 10,000 new dengue infected cases were reported in 2018 which dramatically increased to 101,354 cases and 164 deaths in 2019 [6]. However, the infected cases were declined in 2020 due to improved community awareness, strengthen authority sensitization and services, and facilitated environmental protection measures, moreover 2020 was the next year after the outbreak of COVID-19 during the period all other diseases were sub-diagnosed due to the high number of cases of COVID-19. It was markedly declined in 2020 with 1405 cases and 3 deaths [7,8]. Unfortunately, the cases and deaths were increases to 28,429 and 105 deaths in 2021 which was more than double from 2020. The re-emerging trend of dengue was evident in next year. A total of 60,078 cases and 266 deaths were reported in 2022 which was considered as second largest outbreak of dengue fever in Bangladesh since 2000 [9]. In 2023, a total of 321179 cases from different hospitals and 1705 deaths were recorded that exceeds all previous history of outbreaks in Bangladesh [10].

Dengue is a viral disease caused by an arbovirus belonging to the Flaviviridae family. There are four types of dengue viruses: DENV-1, DENV-2, DENV-3, and DENV-4 [11]. The four serotypes are essential for understanding dengue disease severity, developing vaccines, tracking outbreaks, and informing treatment and diagnosis strategies [12]. In Bangladesh, all four strains are present, with DENV-4 being the most common (41%), followed by DENV-2 (25%), DENV-1 (22%), and DENV-3 (13%) [13]. The viral agents of dengue are transmitted from human to human through the bite of female Aedes mosquitoes, primarily *Aedes aegypti* [12]. These mosquitoes typically bite during the daytime (from dawn to dusk) and breed in areas with standing water, such as puddles, water tanks, containers, and old tires, another vector of dengue in Bangladesh is *Aedes albopictus* more prevalent in rural and suburban areas [2,6,11].

Once infected with the dengue virus, individuals may experience a range of symptoms from mild to severe. Mild cases often include fever, headache, muscle and joint discomfort, nausea, vomiting, and loss of appetite. These patients usually do not require hospitalization and can recover with supportive treatment at home or in an outpatient setting [14]. To confirm dengue and differentiate it from other fever such as Malaria, Chikungunya, Typhoid Fever, Leptospirosis Influenza, Japanese Encephalitis, or COVID-19 -causing illnesses, patients should undergo diagnostic tests such as NS1 antigen and DENV-specific IgM tests [15].

However, the dengue prevalent divisions of Bangladesh are Dhaka, Chattogram and Khulna. Unfortunately, the other remaining divisions including Mymensingh have also reported the dengue incidence [16]. Mymensingh division is closely bordered with Dhaka division which has been recognized as highest prevalent zone in Bangladesh, this region is most at risk for dengue. In addition, the presumptive patients are also coming to Mymensingh Medical College Hospital (MMCH), Mymensingh from neighboring divisions. To the best of our knowledge one study is available with dengue patients at a hospital in a non-endemic zone of Bangladesh, where authors investigated the severity of dengue [17]. Based on growing risk and public health burden, the present authors felt the necessary to determine the prevalence and associated factors of dengue among suspected patients with dengue admitted at a tertiary level hospital in Mymensingh region of Bangladesh.

## Methods

### Ethics statement

We followed all rules and regulations of the Institute of Biological Science (IBSc), University of Rajshahi, Bangladesh, and IBSc approved this study and provided an ethical clearance letter (Memo No. 37(21)/320/IAMEBBC/IBSc). Prior to gathering data, we obtained permission from Mymensingh Medical College Hospital, Mymensingh, Bangladesh, and we had extensive conversations with our selected participants regarding the aim of our research, and we took informed written consent from all participants, except for patients at aged<18years, their written consent was taken from their legal guardian.

### Study setting

This epidemiological cross-sectional study was conducted at the inpatient department of medicine, MMCH, Mymensingh. MMCH is a tertiary level government hospital in Mymensingh region.

### Data collection procedure

Data was collected from June 2023 to December 2023 by the first author (Dr. Saiful), he was the duty doctor at dengue unit, a special ward of the inpatient department (IPD) of medicine unit of MMCH. From June to September is the monsoon season in Bangladesh. The heavy rainfall with high temperatures and humidity during the season make ideal breeding conditions for Aedes mosquitoes, which transmit dengue [18]. During the data collecting period all suspected dengue patients (381) admitted at dengue unit were considered as sample for the study. Patients were interviewed using semi-structured questionnaire through face to face at the beginning of admission and collected their socio-demographic, behavior and environmental information. Subsequently, the suspected patients were underwent dengue tests, and test results were collected from hospital records as dengue positive or negative. MMCH has special arrangements like high dependence unit (HDU) and intensive care unit (ICU) for very severe dengue cases who had lower GCS scores.

### Measurement of dengue

The patients were recruited as a presumptive dengue fever if they had an oral temperature of at least 100.4°F, with at least two of the dengue sign and symptoms such as nausea and vomiting, rash, body aches, joint pain or any relevant warning signs [13]. After recruitment, dengue infection was confirmed by a positive result of NS1 dengue antigen test if the blood sample taken within 7 days of the onset of fever, and IgM antibody test if the blood sample taken after 7th day of symptoms for identification of new case. Those patients who had both IgM and NS1 were negative but IgG was positive who identified as previous dengue infected patients. The enzyme-linked immunosorbent test (ELISA) technique was used to identify the NS1 antigen, anti-dengue IgM antibodies for diagnosis. NS1 antigen, anti-dengue IgM were interpreted as positive or negative reports only. Accuracy and completeness of the data were checked thoroughly on the same day of data collection. Admitted patients were suspected by MMCH, and all relevant testes for confirming as dengue patients were done by dengue unit, MMCH.

### Outcome variable

Dengue was the outcome variable of the study, and it was measured by NS1 dengue antigen test or IgM antibody test. The positive result of these tests was considered as having dengue (yes, code = 1), and negative result as not having dengue (no, code = 0).

### Independent variables

Age, gender, education, residence, presence of water container around the house, regular cleaning of draining system at living place, use of mosquito net during sleeping at day time, travel history to Dhaka in last two weeks preceding the survey, construction site around the living place were considered as possible predictors of dengue.

### Statistical analysis

Collected data were analyzed using the statistical package for the social sciences (SPSS) software (IBM version 25). Descriptive statistics were used to summarize the data. The chi-square test/ Likelihood Ratio test (if any cell frequency 0 for not 2X2 tables) and multiple binary logistic regression model were used to identify the associated factors of dengue fever. Variance inflation factor (VIF) was used to measure the amount of multicollinearity among independent variables in the logistic regression model. There is no evidence of multicollinearity problems if the value of VIF lies between 0 and 5 [19]. The logistic regression results were interpreted by adjusted odds ratio (aOR) with 95% confidence interval (CI) (lower and upper values of aOR), and p-value. The levels of significance of p < 0.01 and p < 0.05 were considered.

## Results

Table 1 illustrates the sociodemographic characteristics of the respondents. The highest number of respondents was young adults (age, 20–40 years). More than half participants were males, and majority of the respondents were uneducated or primary level. More than 64% respondents came from urban areas. Among the participants, more than one-third had regular cleaned the draining system at their living place. In this study, only 18.6% respondents used mosquito net during sleeping at day time, and more than half had presence of water containers around their house. A large portion of patients had travel history to Dhaka in last two weeks preceding the survey.

**Table 1:** Characteristics of respondents, and its association with dengue.

Variable	Group	N (%)	Dengue	Chi-square/ Likelihood Ratio	p-value	Variable
Yes	No
Age (year)	<20	54(14.2)	42(77.8)	12(22.2)		
	20-40	186(48.8)	100(53.8)	86(46.2)	120.432^a^	0.001
	≥40	141(37.0)	141(100)	0(0.0)		
Gender	Male	205 (53.8)	140 (68.3)	65 (31.7)	8.322	0.004
	Female	176 (46.2)	143 (81.3)	33 (18.7)		
Education	Uneducated & primary	195 (51.2)	162 (83.1)	33 (16.9)	16.592	0.001
	Secondary	74 (19.4)	50 (67.6)	24 (32.4)		
	Higher	112 (29.4)	71 (63.4)	41 (36.6)		
Residence	Urban	245 (64.3)	204 (83.3)	41 (16.7)	29.015	0.001
	Rural	136 (35.7)	79 (58.1)	57 (41.9)		
Travel history to Dhaka in last two weeks	Yes	238 (62.5)	197 (82.8)	41(17.2)	23.951	0.001
	No	143 (37.5)	86(60.1)	57 (39.9)		
Presence of water container around the house	Yes	197 (51.7)	169 (85.8)	28 (14.2)	28.278	0.001
	No	184 (48.3)	114 (62.0)	70 (38.0)		
Regular cleaning of draining system at the living place	Yes	121 (31.8)	94 (77.7)	27 (22.3)	1.078	0.299
	No	260 (68.2)	189 (72.7)	71 (27.3)		
Construction site around the living place	Yes	245 (64.3)	188 (76.7)	57 (23.3)	2.168	0.141
	No	136 (35.7)	95 (69.9)	41 (30.1)		
Use of mosquito net during sleeping at day time	Yes	71 (18.6)	40 (56.3)	31 (43.7)	14.700	0.001
	No	310 (81.4)	283 (74.3)	98 (25.7)		

**Note:** a, Likelihood Ratio value.

**Fig 1:**
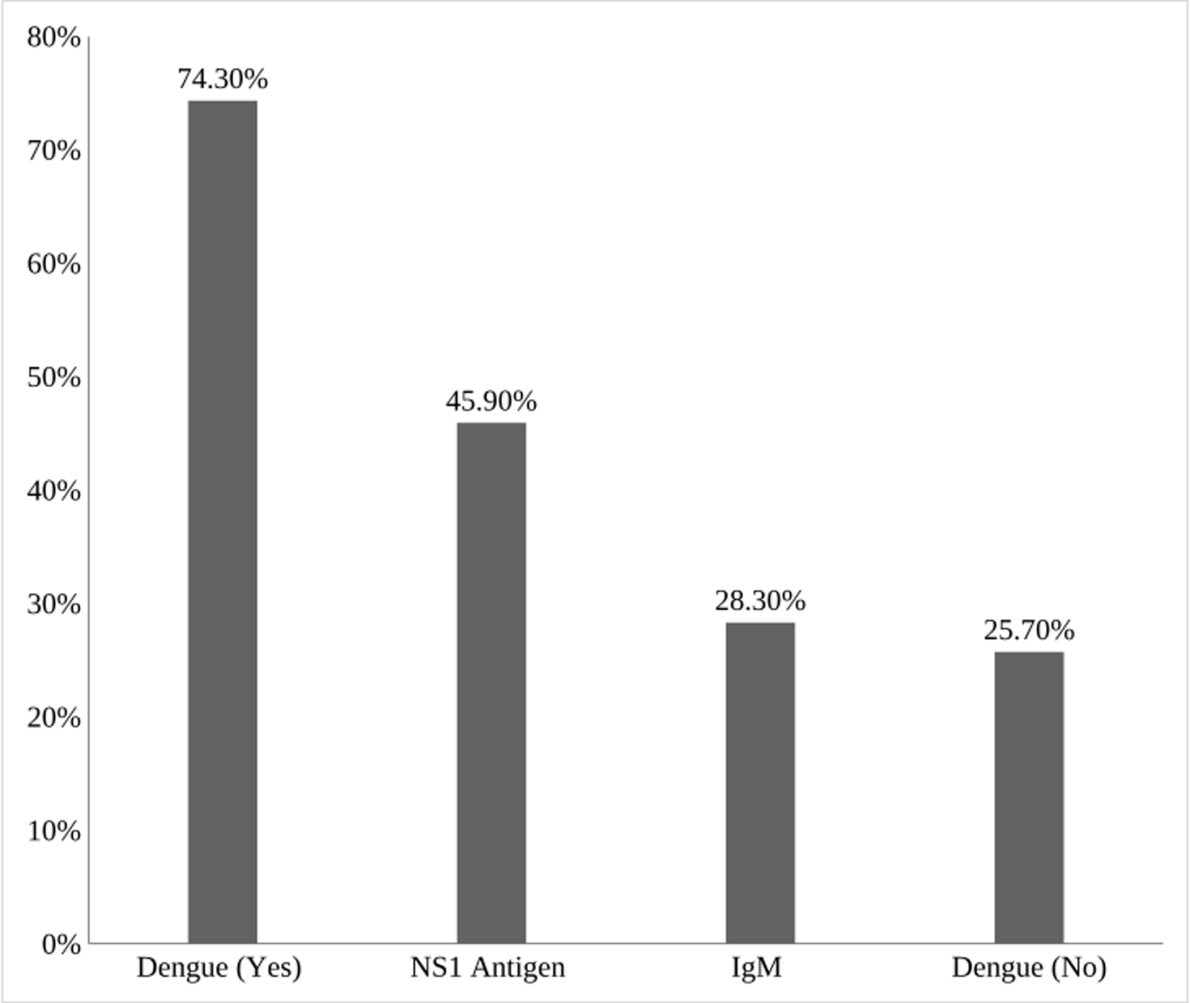
Prevalence of dengue fever confirmed by NS1 and IgM (n = 381).

In this study, out of all suspected dengue patients (381), serological tests (NS1 and IgM) confirmed 283 (74.3%) patients were suffering from dengue. Out of total patients having dengue, 175 (45.9%) was confirmed by NS1 antigen test who came to the hospital within seven days of symptoms whereas 108 (28.3%) of patients were confirmed by dengue specific IgM antibody tests receptively who came after seven days of onset of symptoms (Fig 1).

The chi-square test showed that female had significantly (p < 0.05) higher dengue than male. The uneducated and primary educated respondents were more prone to dengue than their counterparts (p < 0.001). Consequently, urban residence, travel history to Dhaka in last two weeks, and presence of water containers around the house were significantly associated with development of dengue (p < 0.001). Furthermore, use of mosquito net during sleeping at day time had significantly less chance to get dengue (p < 0.001). It was observed that 100% adult (age ≥ 40 years) suspected with dengue were identified as dengue patients, and the likelihood ratio test showed that the association between age and dengue was significant (p < 0.001). Regular cleaning and construction site around the living places were not significantly associated with developing dengue (p > 0.05) (Table 1).

The significant associated factors provided by Chi-square test were considered as independent variables in multiple binary logistic regression model where dengue fever (yes or no) was considered as dependent variable. Though age was a significant associated factor of dengue provided by Likelihood Ratio test, but we did not consider the variable as independent variable in logistic model due to 100% suspected adult (age ≥ 40 years) patients were identified as dengue patients. VIF showed that there was no evidence of multicollinearity problems among independent variables in the model. After controlling the effects of other variables, the model demonstrated that female had more likely to have dengue than male [aOR = 2.08, CI: 1.09-3.93; p < 0.05]. Participants came from urban environment had more chance to have dengue fever compared to rural respondents [aOR = 5.99, CI: 3.09-11.64; p < 0.01]. Participants who had travel history to Dhaka in last two weeks were more likely to get dengue fever compared to participants who did not travel. We found that respondents who did not use mosquito net during sleeping at day time had a 2.7-fold higher change to have dengue fever compared to participants who used the mosquito net at day time [aOR = 2.743, CI: 1.411-5.333; p < 0.01]. Presence of water containers around the house was an important predictor of dengue fever, participants had presence of water container within and around their house were more vulnerable to get dengue fever compared to their counterparts [aOR = 12.00, CI: 5.69-25.29; p < 0.01]. Uneducated or primary educated participants were more likely to get dengue fever than higher educated participants [aOR = 2.83, CI: 1.66-4.85; p < 0.01]. The Negelkerke R^2^-value showed that the model can able to explain the variation of dependent variable by nearly 60% (Table 2). Moreover, the accuracy of the test was measured using the ROC curve. The area under the ROC curve was 0.913, which means that in 91.3% of the cases, the model correctly assigned a higher probability of having dengue fever to the subject who actually got it (S1 Fig).

**Table 2:** Binary logistic regression analysis of factors influencing dengue fever among suspected patients.

Variable	Group	B	p-value	aOR	95% CI for aOR		
Lower	Upper	VIF
Gender	Female Vs Male^R^	0.731	0.025	2.078	1.097	3.934	1.157
Residence	Urban Vs Rural^R^	1.791	0.001	5.996	3.087	11.644	1.245
Travel history to Dhaka in last two weeks	Yes Vs No^R^	2.417	0.001	11.210	5.296	23.725	1.444
Presence of water container around the house	Yes Vs No^R^	2.485	0.002	12.000	5.693	25.293	1.255
Use mosquito net during sleeping at day time	No Vs Yes^R^	1.009	0.003	2.743	1.411	5.333	1.096
Education	Uneducated or primary Vs Higher^R^	1.042	0.001	2.835	1.658	4.848	1.515
	Secondary Vs Higher^R^	0.185	0.559	1.203	0.647	2.237	
	Negelkerke R^2^-value	0.594					

**Note**: B, Regression coefficients; aOR, adjusted odds ratio; CI, Confidence interval; VIF, Variance inflation factor

## Discussion

In this study, 381 suspected patients were enrolled between June and December, 2023 from Mymensingh Medical College Hospital (MMCH), Mymensingh to investigate dengue fever of suspected patients. We found around 74% confirmed cases which showed dengue symptoms that corresponding to serological test results. Based on duration of illness, two types of tests were done for confirmation and management of dengue fever. The NSI antigen test is the gold standard for detecting dengue within seven days of illness and after seven days, the recommended test is IgM antibody test. As the suspected patients were coming to MMCH during the different phases of illness, the hospital ensured its confirmation following both diagnostic methods corresponding to symptoms. We found 45.9% and 28.3% positive dengue through NS1 antigen test, and dengue specific IgM antibody test receptively. An earlier study in Bangladesh showed that positivity rates of 69% for the NS1 antigen test and 65% for the dengue-specific IgM antibody test [20]. In comparison, China reported an NS1 antigen positivity rate of 82.9% and an IgM antibody positivity rate of 49.5% [21] while Myanmar showed NS1 antigen and IgM antibody positivity rates of 67.1% and 83.9%, respectively [22]. Additionally, higher NS1 antigen positivity rates in China may suggest more acute phase infections, whereas the elevated IgM antibody rate in Myanmar indicates a larger proportion of samples collected during the later phase of infection.

As a part of tropical area, the climate of Bangladesh is mainly divided into two monsoons (wet and dry monsoons). Dengue cases are more reported during monsoon months when relative humidity is higher. The higher humidity during the rainy season facilitates the growth and survival of infected mosquitoes for the successful propagation of the virus [19,20]. The dengue can affect people irrespective of gender, residence, education level. We found 205 (53.8%) male patients, of them, 140 (68.3%) had dengue positive. On the other hand, out of 176 (46.2%) female patients, 143 (81.3%) had positive test results indicates that female had significantly higher chance of developing dengue. This might be due to more exposed to household water containers particularly during cleaning process, less use of mosquito net while sleeping at day time. Though another study in Bangladesh found male had higher chances of getting bitten by Aedes mosquito at working place than women [23]. We analyzed the socio-demographic and environmental factors and found more cases from urban area (83.3%). The mosquito breeding places and environment such as construction sites around the house, open waste and dirt, old vehicle tires, uncleaned and open drainage, poor hygiene practice, improper environmental sanitation, clean water containers inside the house, poor mosquito control measures are available in urban areas that increasing the risk of spreading dengue virus through Aedes mosquito. As Bangladesh is rapidly urbanizing country, it favors the risk of spread of dengue among urban visitors and residents [24]. Several studies found similar results which has validated our study [17,23,25–27]. The education and life style factors significantly affect the behavior of individual and families. In our study, we found uneducated and primary educated patients had more dengue (83.1%) than their counterparts. This may indicate the level of knowledge, awareness and attitude towards prevention of dengue. Another study in Caribbean region of Colombia also found similar results [28]. The behavior related another factor we considered in our study which was ‘use of mosquito net while sleeping at day time (dawn to dusk)’. As Aedes mosquito usually bite at day time, we extracted the practice level and found around 18% use rate. Of them, less percentage (56%) of occurrence of dengue was found among mosquito net users indicate its benefits to prevent dengue. A study conducted in Karnataka, India provided similar significant result about the correlation between using mosquito net with reducing dengue epidemics [29]. Like the mentioned factors, history of travel was another crucial factor which was significantly associated with developing dengue. Majority of our study participants (62.5%) had frequent travel history to Dhaka as resourceful capital for job, business, or other opportunities. Dhaka is the worst hit area and the epicenter of dengue outbreak, with more than half of the cases being reported in this megacity. In our study, those who travelled in last two weeks, they had 82.8% dengue positive results. Different studies in Bangladesh showed that travel to epidemic and endemic city like Dhaka can increase the risk of dengue [17, 30]. Subsequently, those who had water containers within and around the living place, they had higher chance to get dengue than their counterparts. The water containers are the suitable place for mosquito breeding thus facilitating dengue transmission opportunity. To prevent the dengue transmission, several studies and available mass media advertisement warn about cleaning of water containers around and within the living places at least every three days’ interval [11,13].

### Limitations

We did not measure the association between severity and its associated factors, poor-rich association, specific prevalence in different geographic locations or districts within and outside the Mymensingh division. We did not analyze the rate of dengue among different occupations. We did not analyze the treatment outcome (deaths and cure rate), and signs and symptoms. These factors would provide potential clues to researchers for further study. We used non-probability sampling for selecting sample which was limited in generalizing the findings.

## Conclusion

Dengue fever is sometimes a severe and debilitating disease that may be caused widespread anxiety, panic, and disruption. This study attempted to determine the prevalence and associated factors of dengue among suspected patients with dengue admitted at hospital to take appropriate steps, interventions, and educate the general public. The main concern is that dengue is rapidly spreading throughout the world, and there is no potential or specific treatment or vaccine. Bangladesh is struggling to aware, inform and educate people to prevent and control of dengue through destroying breeding places, preventing through using mosquito net or repellents, and providing symptomatic treatment and managing complications. As it recently become one of the top causes of hospitalization and death among children and vulnerable individuals, posing a significant challenge to the health-care system. Mass awareness with environmental cleaning, sanitation, regular cleaning of living and working areas, planned infrastructure development, proper garbage and waste disposal systems, and practice of various preventive measures can benefit the people through reducing sufferings and risk of deaths. Moreover, the study recommends strengthening healthcare infrastructure, promoting community-based vector control, developing national dengue control programs, improving public health communication, ensuring affordable access to preventive measures, and collaborating with international organizations for effective dengue management. The overall findings of the study could help physicians and health personnel to detect dengue from clinical suspicion correctly.

## Supporting Information

S1 Data Questionnare.docx Sup-Figure.tiff. (SAV)

S1. File Questionnaire. (DOCX)

S1 Fig Prevalence of dengue fever confirmed by NS1 and IgM (n=381). (TIFF)
