# Supplementary material for: Dengue among suspected patients with dengue admitted at a tertiary level hospital in Mymensingh region of Bangladesh: A hospital-based epidemiological study
Source: PLoS Negl Trop Dis. 2025 Apr 28;19(4):e0013047. doi: 10.1371/journal.pntd.0013047 (PMC12036909; doi:10.1371/journal.pntd.0013047)
Supplement: S1. File — (DOCX) [file pntd.0013047.s002.docx]

**Title: Assessment of obesity and quality of life of Dengue patients**

[Questionnaire]

[Please circle the response by ball point]

| **Part-1: Socio-Demographic and clinical characteristics** | | | | |
| --- | --- | --- | --- | --- |
| Serial no: |  | | | |
| 1 | **Date of admission (This is mandatory):** | | | |
| 2 | **Age (years):**   - <20 - 20-40 - ≥40 | | | |
| 3 | **Gender :**   - Male - Female - Others | | | |
| 4 | **Education:**   - Uneducated - Primary - Secondary - Higher | | | |
| 5 | **Residence:**   - Urban - Rural | | | |
| 6 | **Monthly family income (BDT):** | | | |
| 7 | **Did you have travel history to Dhaka in last two weeks?**   - Yes - No | | | |
| 8 | **Did you have water container around your house?**   - Yes - No | | | |
| 9 | **Did you regular cleaning of draining system at your living place?**   - Yes - No | | | |
| 10 | **Had any construction site around your living place?**   - Yes - No | | | |
| 11 | **Did you use of mosquito net during sleeping at day time?**   - Yes - No | | | |
| 12 | **Features of plasma leakage:**   - Edema - Pleural effusion - Ascites | | | |
| 13 | **Dengue NS1 antigen:**   - Positive - Negative | | | |
| 14 | **Tourniquet test:**   - Positive - Negative | | | |
| 15 | **IgG:**   - Positive - Negative | | | |
| 16 | **IgM:**   - Positive - Negative | | | |
| 17 | **Liver enzymes:**   - ALT:_________[write the value from report] - AST:_________[write the value from report] | | | |
| 18 | **Severity of Dengue (WHO guideline 2009):**   - Dengue without warning signs (Group-A) - Dengue with warning signs (Group-B) - Severe dengue (Group-C) | | | |
| 19 | **Date of discharge (This is mandatory):** | | | |
| **Part-2: Quality of life assessment of dengue patients**  **The quality of life will be assessed by the EQ-5D-3L scale. Under each heading, please tick the ONE box that best describes your health TODAY.** | | | | |
|  |  | ***Day of admission*** | ***Day of discharge*** | ***14 days after discharge*** |
| 22.1 | Mobility:   - I have no problem in walking about - I have some problem in walking about - I am confined to bed |  |  |  |
| 22.2 | Self-care:   - I have no problem with self-care - I have some problem in washing or dressing myself - I am unable to wash or dress myself |  |  |  |
| 22.3 | Usual activities:   - I have no problem with performing my usual activities - I have some problem performing my usual activities - I am unable to perform my usual activities |  |  |  |
| 22.4 | Pain/Discomfort:   - I have no pain or discomfort - I have moderate pain or discomfort - I have extreme pain or discomfort |  |  |  |
| 22.5 | Anxiety/Depression:   - I am not anxious or depressed - I am moderately anxious or depressed - I am extremely anxious or depressed |  |  |  |
| 22.6 | This scale is numbered from 0 to 100 :    - '100' means the best health you can imagine.            -  '0' means the worst health you can imagine.  Mark an 'X' on the scale to indicate how your health is TODAY.  Now, please write the number you marked on the scale in the box below.   - **At day of admission: __________________________** - **Day of discharge: __________________________** - 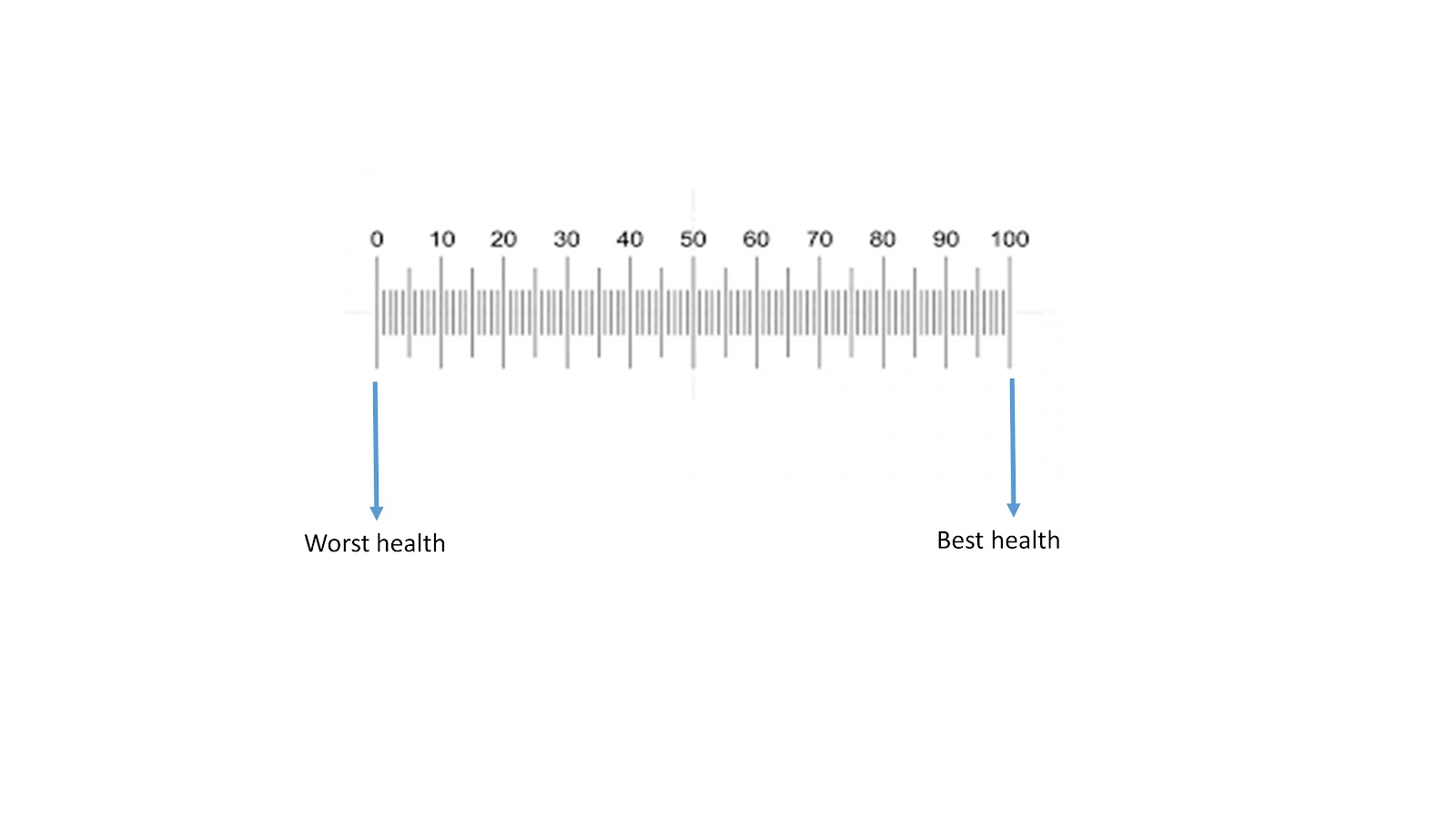**14 days after discharge: __________________________** | | | |

Respondent’s Signature: Data collector’s Signature:
