# Supplementary figures and images for: Dengue among suspected patients with dengue admitted at a tertiary level hospital in Mymensingh region of Bangladesh: A hospital-based epidemiological study
Source: PLoS Negl Trop Dis. 2025 Apr 28;19(4):e0013047. doi: 10.1371/journal.pntd.0013047 (PMC12036909; doi:10.1371/journal.pntd.0013047)

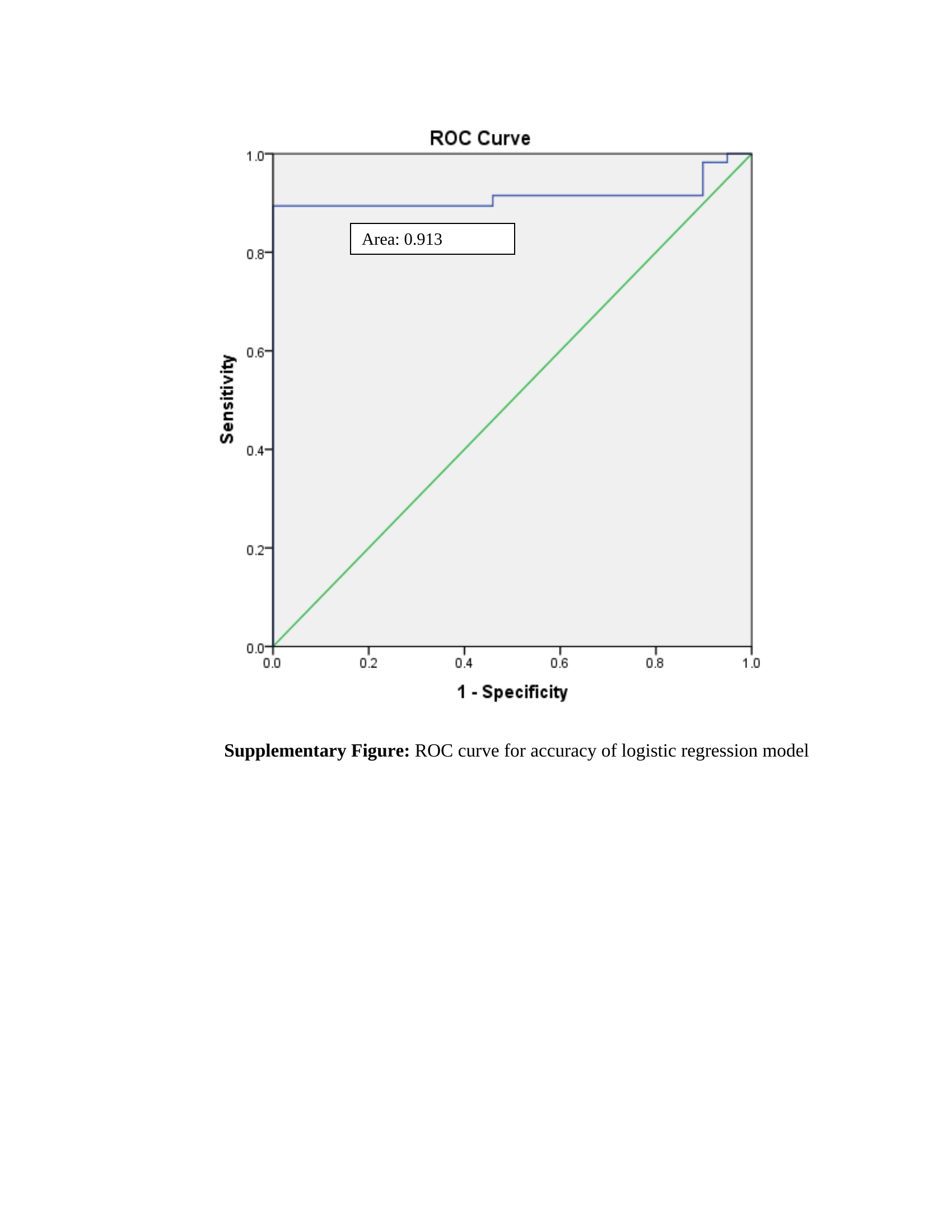

Supplement: S1 Fig — (TIFF) [file pntd.0013047.s003.tiff]
